# The role of selected pre-match covariates on the outcome of One-day International (ODI) cricket matches

**DOI:** 10.17159/2078-516X/2023/v35i1a15012

**Published:** 2023-06-05

**Authors:** K McEwan, L Pote, S Radloff, SB Nicholls, C Christie

**Affiliations:** 1 Department of Human Kinetics and Ergonomics, Rhodes University, Grahamstown, South Africa; 2 Department of Statistics, Rhodes University, Grahamstown, South Africa; 3 Department of Sport, Outdoor and Exercise Science, University of Derby, England

**Keywords:** cricket, match type, toss decision, toss outcome, venue

## Abstract

**Background:**

The identification of key factors that systematically influence a team’s success is important and has led to the application of statistical models in sport. Predicting the outcome of a One Day International (ODI) cricket match, using only pre-match covariates, has been minimally investigated.

**Objectives:**

This research sought to investigate the impact that venue, toss outcome, toss decision, and match type have on the chances of winning an ODI match.

**Methods:**

A total of 1228 men’s international ODI matches were analysed. A logistic regression model was used to identify the significance of these pre-match covariates on the result of the matches.

**Results:**

The results varied across all teams, suggesting that there are individualised factors driving these differences and that generalising the impact pre-match covariates have in every team is unrealistic. New Zealand and India displayed a significant home advantage effect, whereas Australia had a strong tendency towards a significant disadvantage when they won the toss. However, for most teams, toss outcome, toss decision, and match type did not significantly impact the outcome of an ODI match.

**Conclusion:**

New Zealand and Australia were the most predictable teams, whereas South Africa and Pakistan were regarded as unpredictable when pre-match covariates were used to forecast the outcome of their ODI matches.

The identification of factors which influence the likelihood of winning through statistical modelling has grown in popularity.^[1,2]^ Cricket is the second most popular sport worldwide and predicting the match outcome has been exploited in the betting industry; however, the potential predictive factors of cricket matches have not been extensively researched.^[3]^ This may be because of the multifaceted and inconsistent nature of the game.^[4]^ Furthermore, while the sports betting industry is constantly growing (specifically in cricket) and this type of information can be used by bettors, the prediction of the final match outcome may also prove beneficial to team members and coaches.^[5,6]^ It has been suggested that the team management, coach and captain can analyse specific characteristics of cricket matches to predict match performance.^[7]^ In forecasting match results in cricket, factors are categorised under two covariate groups; pre-match, which predicts the outcome prior to the match; and in-play, which predicts the outcome while the match is in progress.^[3]^

Pre-match covariates relate to several aspects that have been identified to impact upon match outcome. Within cricket, these include but are not limited to, home advantage, team strength, toss outcome, toss decision, and match type. The home advantage concept is based on the phenomenon that teams win more than 50% of their matches on home ground.^[8]^ Home advantage has been previously identified in ODI matches,^[9,10],^ particularly for India, South Africa, Australia, Sri Lanka, New Zealand and Pakistan.^[4]^ However, it has been suggested that home advantage in cricket has slowly diminished over recent years.^[2]^ This could be due to the emergence of the new Twenty20 leagues which provide players with more global exposure.^[2]^ The various factors that contribute to home advantage in sports are familiarity with pitch conditions, travel effects, tactics, crowd factors, umpire bias and psychological changes.^[11]^ However, even though each factor has perceptive appeal and slight empirical support, there is no strong evidence to indicate that any of these factors alone, or in combination, determine a home advantage.^[8,12]^

Team strength must be considered and controlled when quantifying the effect of both the home advantage and the coin toss.^[13]^ Team strength is normally based on the International Cricket Council’s (ICC) official ODI rankings; however, it is often criticised as it uses an ad-hoc points system entirely based on matches won and lost.^[14]^ Research has proposed, but has not yet implemented, a weighted TeamRank (WTR) method which increases points given to a team when they gain a win against a stronger team as opposed to a win against a weaker team.^[14]^ Team strength can also be internally quantified by calculating the quality of each player’s bowling and batting capacity.^[10]^ This information could influence the tactical decisions of a team, depending on the opposition’s dominant ability.^[10]^

The toss decision, weather conditions, and match type are all considered when determining the magnitude of the advantage that winning the toss offers.^[1,15]^ Investigating the effect of the coin toss on ODI matches, particularly the debate regarding its removal, was completed recently in English county cricket.^[16]^ There is no evidence to suggest that teams gain a winning advantage because of winning the toss in ODIs.^[4,9,10]^ A small advantage has been seen,^[17]^ but these results were obtained using unreliable statistical means, omitting key statistical procedures, such as odds ratios, confidence intervals, likelihood rest ratios and specific hypothesis testing. Furthermore, a disadvantage of winning the coin toss has been seen in day matches.^[2]^ This could indicate that despite the strategic opportunity the toss provides, more teams are prone to making incorrect toss decisions by over- and/or underestimating their opposition’s strengths and weaknesses.^[2]^ The decision to bat or bowl first, after being given the opportunity through winning the toss, is mainly dependent on the team’s bowling and batting ability.^[11,18,19]^ It has been proposed that fast bowlers prefer to bowl first during day games, as there is more moisture on the pitch.^[18]^ However, it has also been suggested that teams such as India, which generally have a bowling line-up dominated by spinners,^[17]^ prefer bowling second, after the pitch has worn down.^[18]^ Most teams often decide to bat first because of the unpredictability of the second half of the match possibly being influenced by weather conditions.^[4]^ However, batting second could be an advantage because the team can implement an appropriate strategy to win, as they are cognisant of the run chase target.^[10,18]^ Individual venues must also be taken into consideration when making the toss decision, as some pitches favour the side batting first and vice-versa.^[20]^ The weather can also affect the playing conditions and outcome of a match^[13,21]^, with results or run targets changing due to the implementation of the Duckworth-Lewis-Stern (DLS) method.^22]^

The ODI format involves two different match types: day-only and day-night game.^[18]^ The coin toss is seen to be more crucial in day-night matches as playing conditions are considerably different.^[17,18,21]^ During a day-only game, both teams play entirely under natural light, whereas during a day-night game, artificial lighting is used during the evening.^[19]^ It has been suggested that batting second in day-night games is seen as a disadvantage because the artificial light lessens the visibility of the white ball.^[19]^ However, those who bowl second also experience difficulties in the evening as the dew factor causes poor ball grip, which increases the likelihood of bowling inaccuracy.^[13,19]^

Although these pre-match factors influence ODI matches, the modelling of these factors has been minimally investigated. Additionally, existing research has not performed the necessary diagnostic tests; thus, the validity and reliability of their results are compromised. Tests such as the likelihood-ratio test, best model selection (AIC), hypothesis testing, odds ratios, and confidence intervals have not previously been included in research studies. However, it is important that these are considered to ensure a robust study design. Therefore, the purpose of this study was to construct binary logistic regression models for eight teams as part of the ICC, using venue (home/away), toss outcome (win/lose), toss decision (bat first/second) and match type (day/day-night) to predict the outcome of ODI matches. This may assist in determining how these variables influence the game, and if they are significant enough to potentially predict the winner before the match commences.

## Methods

### Sample

A total of 1228 men’s international ODI matches involving South Africa, Australia, New Zealand, Sri Lanka, England, Pakistan, West Indies, and India played between January 2007 and July 2017, were selected for analysis. These eight competitors were selected as a sample of convenience as they have regularly competed and are full members of the ICC. The period of ten years was chosen to enable an appropriate number of observations to be collected to perform reliable logistic regression analyses. Additionally, the ODI limited-overs form was selected as (i) there are more win/loss results generated compared to test matches and (ii) it provides a more balanced schedule of regular fixtures between teams. Female teams were not considered to have played significantly fewer ODIs compared to the men, within the selected time period. Furthermore, test and T20 matches were also excluded.

### Procedure

Data regarding four predictors (venue, toss outcome, toss decision, and match type) and one response variable (match outcome) were collected manually from match scorecards, which are openly available via the ESPN Cricinfo website. Match type referred to whether a match was a day or day-night game. To control for extraneous factors, data went through a filtration process where matches were excluded if (1) the result ended in a tie or no result, (2) the Duckworth-Lewis/Duckworth-Lewis-Stern method was used, (3) the match took place at a neutral venue, or (4) the umpires were both local. The statistical software R^®^ (Version 1.0.153) was used for all data analysis processes.

### Statistical analysis

A logistic regression model was constructed to identify the significance of the pre-match covariates (Venue [home, away], Toss outcome [win, loss], Toss decision [bat first, bat second], and Match type [day, day-night]), both collectively and individually, for each cricket team in relation to the response variable (Match outcome [win, lose]). No multi-comparison correction tests were done and each country was individually critiqued and analysed.

The Wald chi-squared statistic (z^2^), which is a χ^2^ distribution with one degree of freedom, was used to assess whether the individual variables significantly impacted upon match outcome (z^2^ > 3.84). The odds ratio was also calculated to predict the likelihood of winning an ODI while controlling for the other predictors in the model. Additionally, after fitting the model for each cricket team, a likelihood ratio test was used to assess whether the variables as a whole significantly impacted match outcome. All significance thresholds were set at 0.05. The best fitted model was selected by identifying the model which had the lowest Akaike information criterion (AIC) value. This was determined using a stepwise elimination algorithm in both directions (i.e. forward, and backward).

McFadden pseudo R^2^ was calculated to measure the predictive value of the model. Values between 0.2 to 0.4 were considered satisfactory (below 0.2 was considered poor). Receiver operating characteristic (ROC) curves were also used to visually demonstrate the trade-off between sensitivity and specificity and the area under the ROC curve (AUROC) was calculated to give a measure of predictive power. The fitted logistic regression curve, represented by Equation 1, was used to estimate probabilities for given individual scenarios.


(1) $$p_{i}=\frac{e^{\left(\beta_{0}+\Sigma_{j=1}^{k}\beta_{j}x_{ij}\right)}}{1+e^{\left(\beta_{0}+\Sigma_{j=1}^{k}\beta_{j}x_{ij}\right)}}$$

Scenario-based predictions were also made, for example purposes, using the models which have the highest predictable power. Only past matches played over the same ten-year period between the teams involved in the scenarios were included in the generalised linear model (glm) to make the predictions and demonstrate the overall usability of the process.

## Results

### Logistic regression models output

All teams showed positive estimate values, indicating that playing at home had a positive effect on the likelihood of winning an ODI game (Table 1). However, only Australia (z2 = 10.32; p = 0.0013), New Zealand (z2 = 9.63; p = 0.0019) and India (z2 = 5.40; p = 0.0202) had significant positive relationships. The odds ratio of Australia, New Zealand and India indicate that they were 2.85, 3.31 and 2.11 times more likely to win an ODI during home games than away games (Odds ratio for remaining teams ranged between 1.34 and 1.92). All teams (except India) had negative estimate values indicating that playing day-night games had a negative effect on the likelihood of winning an ODI game. However, no significant (p < 0.05) relationship between match type and the outcome of an ODI match were found across all teams (Table 1).

All teams (except the West Indies) showed positive estimate values indicating that winning the toss negatively affected the likelihood of winning an ODI game (Table 2). However, none of these relationships was significant, except Australia, which showed a strong tendency towards statistical significance (z2 = 3.82; p = 0.0506). Australia’s respective odds ratio of 0.54, indicates that they were the least likely team to win an ODI when they won the toss (Table 2). South Africa, England, Pakistan, West Indies, and India showed positive estimate values whereas Australia, New Zealand and Sri Lanka had negative estimate values. However, no significant (p < 0.05) relationship between the toss decision and the outcome of an ODI match was found across all teams (Table 2). The variables “venue”, “toss outcome”, “toss decision” and “match type” can collectively be used to forecast the outcome of an ODI match for Australia (χ2=17.49; p = 0.002) and New Zealand (χ2=13.77; p = 0.008).

### Best model selection

Using only the variable ‘venue’ can be used to best predict the outcome of an ODI game for England, Sri Lanka, West Indies, and India. Additionally, a model using ‘venue’ and “toss outcome” best predicts the outcome for Australia whereas ‘venue’ and ‘match type’ best predict the outcome for New Zealand. None of the variables accurately predict the outcome of an ODI game for South Africa and Pakistan (Table 3).

### Overall predictive analysis

Table 4 demonstrates that all teams are unable to strongly classify a win or a loss using the pre-match covariates. The most predictable teams are New Zealand (R2 = 0.077; AUROC = 0.69) followed by Australia (R2 = 0.065; AUROC = 0.67).

### Example-based predictive analysis

Winning the toss has a significant negative effect on Australia winning an ODI against South Africa (z^2^ = 5.28; p = 0.022). The odds of Australia winning an ODI game against South Africa is only 0.037 times more likely when winning the toss than when losing the toss (Table 5).

Home advantage is significantly seen in New Zealand and positively affects the likelihood of winning an ODI match against Australia (z^2^= 6.68; p = 0.010). When New Zealand play at home, they are 36.33 times more likely to win an ODI against Australia than when they play away in Australia (Table 6).

The following scenarios (a) and (b) were predicted using models constructed in Tables 5 and 6. These models were based on Australia and New Zealand respectively, since they showed the highest predictive ability (Table 3).

- Australia is playing South Africa at home and win the toss. Australia chooses to bat first and the match format played is a day-night game. What is the probability that Australia will win this match? ***Probability = 0.312= 31.2%***
- New Zealand are playing Australia at home and win the toss. New Zealand bat first and the match format played is a day-night game. What is the probability that New Zealand will win this match? ***Probability = 0.569= 56.9%***

## Discussion

Only Australia (z^2^ = 10.32; p = 0.0013), New Zealand (z^2^ = 9.63; p = 0.0019) and India (z^2^ = 5.40; p = 0.0202) present a significant (p < 0.05) positive relationship with playing at home and winning an ODI match (Table 1). This may be because teams must travel in an eastward direction to play at these venues. Research has shown that eastward travel correlates with a reduction in sports performance compared to westward travel.^[23]^ The lack of significant home advantage in most teams could be due to the increased awareness concerning the effects of travel on performance. Thus teams travel to away venues earlier, allowing more time to alleviate the adverse effects of jet lag on performance.^[23]^ One can only speculate that the home advantage found in India, who are known for their world-class spin bowlers,^[17]^ could be as a result of their home pitch curated toward favouring their bowling strength.^[18]^ ‘Venue’ is found to be an influential variable for Australia, New Zealand, England, Sri Lanka, West Indies, and India concerning the outcome of an ODI match (Table 3). As ‘venue’ does not influence South Africa and Pakistan, one could consider these countries as neutral venues for world-class tournaments; however, further research is needed regarding this standpoint. Furthermore, it needs to be noted that Pakistan played most of their home games in the United Arab Emirates for most of the years over the data collection period. Because of the ‘consistency’ of playing their ‘home games’ at the same venue, as well as the fact that certain conditions could be controlled by the team (i.e. pitch conditions), means that this may not have made a massive difference.

Home advantage may also be dependent upon specific opposition teams.^[4,9]^ This is evident with Australia, where a significant overall home advantage is seen (Table 1); however, no significant home advantage against South Africa is observed (Table 5). Psychologically, a home advantage may have an impact on player positivity^[12]^, which could be the case with Australia, New Zealand and India, impacting upon performance in relation to specific venues. Additionally, due to the increase in international Twenty/20 tournaments, players are more accustomed to playing away, potentially reducing the global effect of home advantage.^[2]^ Home advantage, however, cannot be generalised for all cricket teams and formats of the game and thus warrants future research regarding the impact of home advantage on performance.^[9]^ Lastly, pitch preparation could be another reason for the loss of home-ground advantage. Pitch preparation for the shorter formats of the game favours high-scoring matches and as a result, annuls the advantage of teams manipulating the pitch to suit their bowlers.

Winning the toss does not give any statistically significant advantage towards the outcome of ODIs (Table 2).^[4,9,10]^ However, Australia has a substantial disadvantage of winning the toss, suggesting that their toss decision is poorly chosen. As most teams have a non-significant negative relationship with the toss, the debate as to whether the toss should be removed or not could be disputed. Furthermore, no significant evidence exists regarding winning and the decision of a team choosing bat or bowl first, which could suggest that the toss decision may not impact ODI match outcome (Table 2).^[9,10,18]^ From a tactical standpoint, it may be beneficial for teams to be cognisant of the relationship their opposition has with batting first to make a more informed decision if the toss is won. For example, this knowledge could be implemented against teams, such as Australia, New Zealand, and Sri Lanka, who appear to have a minor negative relationship with batting first (Table 2).

All teams, except India, had a negative non-significant relationship with day-night matches and winning an ODI game (Table 1), which correlates with past research.^[4]^ This could mean that specific teams are slightly hindered by the adverse effects of the artificial lighting (visibility) and dew on the ball (affecting grip) during the night session.^[13,17,18]^ A possible resolution is to introduce more night training sessions, which could allow players to adapt to the different conditions experienced between the two match types.

### Predictive ability

All team models are of poor predictive power (Table 4); however, the predictive value for each team can be increased by using their respective best GLM models with the lowest AIC (Table 3). Using only the most influential variables in the models would result in more accurate predictions. This was not investigated in the analyses though as it is beyond the scope of this research paper. Of all the teams, New Zealand and Australia are the most predictable when using the venue, toss outcome, toss decision, and match type as the factors to forecast the outcome of ODIs. South Africa and Sri Lanka provide no evidence of strong relationships to any of the pre-match covariates, which may imply that they are the least predictable teams (Table 4). These findings are evident when examining the area under the curve (AUC); New Zealand and Australia have a higher AUC compared to South Africa, Sri Lanka, and Pakistan. This could be because Australia and New Zealand, over the 10-year data collection period, had a more stable team structure and playing style. Furthermore, they may have had access to data, analysts and technology that assisted in decision-making processes for specific match conditions, i.e. more informed decisions compared to other cricketing nations at the time.

Australia has a significant, negative relationship (z^2^ = 5.28; p = 0.022) with winning the toss and winning an ODI against South Africa (Table 5), which could explain the small winning probability of 31.17% seen in scenario (a). Additionally, Australia is seen to be the least likely team to win an ODI when they win the toss than when they lose the toss (odds ratio = 0.54) (Table 2). This toss disadvantage could indicate that despite the strategic opportunity the toss provides, Australia is mostly prone to making incorrect toss decisions,^[2]^ especially when they play against South Africa. New Zealand, however, has an extreme home advantage (z^2^= 6.68; p = 0.010) when competing against Australia (Table 6). This correlates with home games for New Zealand, where playing at home has a significant positive effect on the likelihood of winning an ODI match (z^2^ = 9.63; p = 0.0019; Table 1).

### Limitations

The overarching venue of a country’s origin in relation to the opposition (Home/Away) was recorded, with specific individual venues within each country not being considered within the analysis. This becomes an important point to consider, as despite the respective team playing within their own country’s boundaries, specific stadia may have diverse features (e.g. pitch slope), weather conditions (e.g. the area of the location has a higher propensity to rain or high winds), and pitch/outfield types (e.g. fast/slow). The scenarios constructed should also be interpreted with caution, as despite these being the ‘best’ examples, all team models overall were of poor predictive power. Lastly, the data was collected over a 10-year period through which team form, players and coaches would have changed. However due to the large sample size of the investigation, as well as random selection, the risk of bias is reduced as well as the fact that logistic regression does not depend on normality. This is an argument that would limit all team sports studies.

### Future directions

Future qualitative research via questionnaires and interview-based approaches is arguably warranted to ascertain the athlete’s perspective regarding the impact pre-match covariates has upon team/individual performance and pre-match preparation. Additionally, it would be beneficial to make use of binary logistic regression, in conjunction with dynamic logistic regression, during the match as it is probable that this may provide a more accurate prediction of match outcome based upon ongoing events (e.g. deliveries remaining, wickets taken). Lastly, this research could be replicated for other formats of the game (i.e. tests and Twenty20) to ascertain whether (1) the use of pre-match covariates holds a meaningful predictive power or (2) the toss decision positively or negatively impacts match outcome for the specific opposition (potentially enhancing a captain’s ability to make a more informed batting/bowling decision) within these formats. To conclude, although not in the scope of this study, future research should also consider additional pre-match covariates such as team composition (bowlers, all-rounders, batters) venue, match type and toss decision, as well as other formats (T20, Test). Further studies could replicate the use of the current method while adjusting the study design based on specifically selected pre-match covariates and match format.

## Conclusion

Binary logistic regression models were used to investigate the effects of venue, toss outcome, toss decision, and match type on winning an ODI match for eight major cricket-playing nations. Varying results were found between the nations concerning each discrete pre-match covariate. This could mean that there are individualised factors driving these differences, therefore generalising the impact that pre-match covariates have in every team is unfeasible. Home advantage is present in ODI cricket; however, significance was only found for Australia, New Zealand and India. Australia had a strong tendency towards a significant disadvantage when winning the toss. However, for most teams, toss outcome, toss decision, and match type did not significantly impact the outcome of an ODI match. New Zealand and Australia were found to be the most predictable teams, whereas South Africa and Pakistan can be regarded as unpredictable when pre-match covariates are used to forecast the outcome of their ODI matches.

## Figures and Tables

**Table 1:** Summary of logistic regression model outputs for variable ‘Venue’ and ‘Match Type’

Team	Venue					Match Type				

	Estimate	z-value	z^2^	p-value	Odds Ratio	Estimate	z-value	z^2^	p-value	Odds Ratio
**South Africa**	0.41	1.17	1.36	0.243	1.50	−0.24	−0.66	0.43	0.511	0.79
**Australia**	1.05	3.21	10.32	0.0013*	2.85	−0.15	−0.40	0.16	0.687	0.86
**New Zealand**	1.20	3.10	9.63	0.0019*	3.31	−0.77	−1.79	3.19	0.074	0.49
**England**	0.51	1.54	2.37	0.124	1.66	−0.13	−0.33	0.12	0.741	0.88
**Sri Lanka**	0.51	1.52	2.30	0.129	1.66	−0.15	−0.44	0.20	0.659	0.86
**Pakistan**	0.29	0.78	0.61	0.435	1.34	−0.22	−0.54	0.29	0.590	0.80
**West Indies**	0.65	1.13	1.28	0.259	1.92	−0.21	−0.34	0.11	0.735	0.81
**India**	0.75	2.32	5.40	0.0202*	2.11	0.065	0.19	0.04	0.850	1.067

* indicates statistical significance (p <0.05)

**Table 2:** Summary of logistic regression model outputs for variable ‘Toss Outcome’, and ‘Toss Decision’

Team	Toss Outcome					Toss Decision				
	
	Estimate	z-value	z^2^	p-value	Odds Ratio	Estimate	z-value	z^2^	p-value	Odds Ratio
**South Africa**	−0.20	−0.55	0.30	0.586	0.82	0.14	0.38	0.14	0.707	1.15
**Australia**	−0.61	−1.96	3.82	0.0506	0.54	−0.28	−0.86	0.73	0.392	0.76
**New Zealand**	−0.13	−0.34	0.11	0.736	0.88	−0.13	−0.34	0.11	0.737	0.88
**England**	−0.019	−0.051	0.003	0.959	0.98	0.065	0.18	0.031	0.860	1.067
**Sri Lanka**	−0.33	−1.03	1.067	−0.302	0.72	−0.26	−0.83	0.69	0.408	0.77
**Pakistan**	−0.22	−0.55	0.31	0.581	0.80	0.54	1.33	1.76	0.185	1.71
**West Indies**	0.29	0.61	0.37	0.543	1.33	0.45	0.97	0.93	0.335	1.57
**India**	−0.10	−0.31	0.094	0.758	0.90	0.38	1.15	1.32	0.252	1.46

**Table 3:** Summary of results using a stepwise elimination method

Team	Best model	Lowest AIC value
South Africa	Null	197.11
Australia	Venue + Toss outcome	260.41
New Zealand	Venue + Match type	172.55
England	Venue	233.96
Sri Lanka	Venue	218.63
Pakistan	Null	194.32
West Indies	Venue	123.70
India	Venue	228.20

AIC, Akaike information criterion

**Table 4:** McFadden Pseudo R^2^ and area under the RORC curves for each cricket team

Team	McFadden pseudo R^2^	Predictive value	AUROC	Predictive power
South Africa	0.014	Poor	0.58	Worthless
Australia	0.065	Poor	0.67	Poor
New Zealand	0.077	Poor	0.69	Poor
England	0.025	Poor	0.60	Poor
Sri Lanka	0.012	Poor	0.58	Worthless
Pakistan	0.012	Poor	0.58	Worthless
West Indies	0.038	Poor	0.63	Poor
India	0.031	Poor	0.62	Poor

McFadden pseudo R2 values below 0.2 were considered poor. AUROC, area under the receiver operating characteristic curve.

**Table 5:** Logistic regression model for Australia vs South Africa (n = 25)

Variable	Estimate	z-value	z^2^	p-value	Odds ratio
Intercept	−0.71	−0.48	0.23	0.635	0.49
Home	1.41	1.30	1.70	0.193	4.077
Win Toss	−3.31	−2.30	5.28	0.022*	0.037
Bat First	1.84	1.36	1.85	0.174	6.26
Day Night	−0.016	−0.013	0.001	0.990	0.98

* indicates statistical significance (p <0.05)

**Table 6:** Logistic regression model for New Zealand vs Australia (n = 28)

Variable	Estimate	z-value	z^2^	p-value	Odds ratio
Intercept	−2.31	−1.46	2.14	0.143	0.099
Home	3.59	2.59	6.68	0.010*	36.33
Win Toss	−0.34	−0.31	0.094	0.760	0.71
Bat First	−1.94	−1.44	2.074	0.150	0.14
Day Night	1.28	0.90	0.82	0.367	3.58

* indicates statistical significance (p <0.05)

## References

[1] Bandulasairi A (2008). Predicting the Winner in One Day International Cricket. MSME.

[2] Khan M, Shah R (2015). Role of External Factors on Outcome of a One Day International Cricket (ODI) Match and Predictive Analysis. Int J Adv Res Comput Commun Eng.

[3] Shah P, Shah M (2015). Predicting ODI Cricket Result. J Hosp Tour Manag.

[4] Jayalath KP (2017). A machine learning approach to analyze ODI cricket predictors. JSA.

[5] Asif M, McHale IG (2016). In-play forecasting of win probability in One-Day International cricket: A dynamic logistic regression model. Int J Forecast.

[6] Kapadia K, Abdel-Jaber H, Thabtah F (2022). Sport analytics for cricket game results using machine learning: An experimental study. Applied Computing and Informatics.

[7] Passi K, Pandey N Increased prediction accuracy in the game of cricket using machine learning. arXiv.1804.04226.

[8] Allen MS, Jones MV (2014). The ‘home advantage’ in athletic competitions. Current directions in psychological science.

[9] De Silva BM, Swartz TB (1997). Winning the coin toss and the home team advantage in one-day international cricket matches. Aust N Z J Stat.

[10] Allsopp P, Clarke SR (2004). Rating teams and analyzing outcomes in one-day and test cricket. J R Stat Soc A.

[11] Morley B, Thomas D (2005). An investigation of home advantage and other factors affecting outcomes in English one-day cricket matches. J Sports Sci.

[12] Bray SR, Widmeyer NW (2000). Athletes' Perceptions of the Home Advantage: An Investigation of Perceived Causal Factors. J Sport Behav.

[13] Sood G, Willis D (2016). Fairly Random: The Impact of Winning the Toss on the Probability of Winning. arXiv.

[14] Daud A, Muhammad F, Yoshida T, Kou G, Skowron A, Cao J, Hacid H, Zhong N Ranking Cricket Teams through Runs and Wickets. Active Media Technology AMT 2013 Lecture Notes in Computer Science.

[15] Dawson P, Morley B, Paton D (2009). To bat or not to bat: An examination of match outcomes in day-night limited overs cricket. J Oper Res Soc.

[16] Wu A (2015). Cricket Australia may follow ECB example and scrap coin toss. The Tonk.

[17] Saikia H, Bhattacharjee D (2010). On the Effect of Home Team Advantage and Winning the Toss in the Outcome in T20 International Cricket Matches. J Sci Technol.

[18] Bhaskar V (2009). Rational adversaries? Evidence from randomizes trials in the game of cricket. Econ J.

[19] McGinn E (2013). The effect of batting during the evening in cricket. J Quant Anal Sports.

[20] Leamon N Arguing the toss. All Out.

[21] Forrest D, Dorsey R (2008). Effect of toss and weather on County Cricket Championship outcomes. J Sports Sci.

[22] Stern SE (2016). The Duckworth-Lewis-Stern method: extending the Duckworth-Lewis methodology to deal with modern scoring rates. J Oper Res Soc.

[23] Lee A, Galvez JC (2012). Jet Lag in Athletes Sports Health.

